# Body Conscious? Interoceptive Awareness, Measured by Heartbeat Perception, Is Negatively Correlated with Self-Objectification

**DOI:** 10.1371/journal.pone.0055568

**Published:** 2013-02-06

**Authors:** Vivien Ainley, Manos Tsakiris

**Affiliations:** Department of Psychology, Royal Holloway University of London, United Kingdom; University of Ulm, Germany

## Abstract

**Background:**

‘Self-objectification’ is the tendency to experience one's body principally as an object, to be evaluated for its appearance rather than for its effectiveness. Within objectification theory, it has been proposed that self-objectification accounts for the poorer interoceptive awareness observed in women, as measured by heartbeat perception. Our study is, we believe, the first specifically to test this relationship.

**Methodology/Principal Findings:**

Using a well-validated and reliable heartbeat perception task, we measured interoceptive awareness in women and compared this with their scores on the Self-Objectification Questionnaire, the Self-Consciousness Scale and the Body Consciousness Questionnaire. Interoceptive awareness was negatively correlated with self-objectification. Interoceptive awareness, public body consciousness and private body consciousness together explained 31% of the variance in self-objectification. However, private body consciousness was not significantly correlated with interoceptive awareness, which may explain the many nonsignificant results in self-objectification studies that have used private body consciousness as a measure of body awareness.

**Conclusions/Significance:**

We propose interoceptive awareness, assessed by heartbeat perception, as a measure of body awareness in self-objectification studies. Our findings have implications for those clinical conditions, in women, which are characterised by self-objectification and low interoceptive awareness, such as eating disorders.

## Introduction

Being self-aware is central to what it means to be human. Not only are we aware of ourselves from a first-person perspective (the position from which ‘I’ perceive the world) but we are also able to consider ourselves from a third-person perspective, as if we were spectators standing outside our bodies and experiencing ourselves as the objects of our own thoughts [1]–[3]. Awareness of self is important for normal experience but is also crucially altered in a number of clinical conditions. Excessive self-focus has been linked to negative affect, anxiety and depression [4]. For example, women who are preoccupied with how their bodies appear from a third-person perspective are vulnerable to a number of mental health conditions, including eating disorders, depression and sexual dysfunction [5]. Anorexia nervosa has been associated with a distorted sense of bodily self, as seen from a third-person perspective [6], as well as with a reduced ability to attend to internal bodily cues of hunger and satiety [7]. Within psychology, awareness of self has been studied from two contrasting perspectives, which have seldom been combined. Social psychology, has concentrated on the affective and cognitive effects of self-focus, manipulated, for example, by looking in a mirror [8] or thinking about self-relevant information [9]. Neuroscientific research, in contrast, has researched the self from the perspective of interoception, which refers to signals arising from within the body [10]–[11]. The purpose of the present study is to bring together these aspects of the self, which have previously been studied independently.

In social psychology, the study of ‘objective self-focus’ was developed as part of a model of self-regulation and affect [12] in which, when the individual's attention is focused inward, the self becomes the object of its own thoughts and perceptions [13]. It is assumed that people compare this perceived self against a salient ideal and attempt to reduce the discrepancy between the two [14]. Self-focus is thus seen as inherently aversive, because the real and desired self are seldom perfectly congruent [14]. Theories of self-focus distinguish between ‘private self-consciousness’, which is the tendency to reflect continually on inner thoughts, sensation and feelings, and ‘public self-consciousness’, in which the individual is concerned with how his or her self is perceived by others [14]. The most commonly used measure of self-focus is the Self-Consciousness Scale [15].

Within this tradition, Fredrickson and Roberts proposed ‘objectification theory’ [16]. They hypothesised that prevailing cultural attitudes, which treat women's bodies as objects for men's gratification, predispose women to value their bodies in terms of physical attractiveness, while men, by contrast, esteem their own bodies for physical effectiveness. Women may come to internalise this objectification and consequently adopt an observer's perspective as the primary view of their physical selves. Women who self-objectify persistently attend to, and monitor, the outward appearance of their bodies. Self-objectification is proposed as an important causal factor in women's mental ill-health, leading to body shame, anxiety and eating disorders, as well as being a potential precursor to depression and sexual dysfunction [16]. Arguing that self-objectification directs attentional resources to the body as perceived from the outside, Fredrickson and Roberts [16] suggested that self-objectification accounts for the relative insensitivity of women to their own internal bodily cues, which has been reported in studies of interoception [17]–[18].

To measure trait self-objectification, Fredrickson and colleagues developed the Self-Objectification Questionnaire [19] and operationalised state self-objectification by requiring participants to wear either a swimsuit (high state objectification) or a loose sweater (low state objectification). Participants in the swimsuit condition were observed to eat smaller amounts of the cookies that they were asked to sample during the experiment [19]. The authors argued that further research would find links between self-objectification and eating disorders, mediated either by body shame [20] or by a lack of attention to ‘internal bodily states’. A number of studies have confirmed the first part of this prediction, establishing the mediating effects of body shame [5]. In self-objectification research, however, finding valid and reliable measures for the ‘awareness of internal bodily states’ has proved problematic. The Body Consciousness Questionnaire and in particular the Private Body Consciousness subscale [21], has been widely used as a measure of inner bodily awareness in this field. However, a review of the objectification literature [5], found little evidence for its role in mediating between self-objectification and cognition, eating disorders, negative affect or depression [22]–[24]. Some authors have concluded that internal body awareness has been mis-measured and mis-conceptualised in the objectification literature [25]. A recent review identified 39 self-report, body awareness measures [26].

Few studies in objectification research have attempted to employ non-questionnaire-based measures of body awareness, such as behavioural and neurophysiological measures. Eshkevari and colleagues used the rubber hand illusion as a psychophysiological measure of body awareness [27]. In this illusion, when a person's real hand is hidden and replaced with a prosthetic, synchronous stroking of the two hands causes an illusion of ownership of the fake hand [28]. Scores on the Self-Objectification Questionnaire significantly predict the extent to which participants experience this illusion and thus the malleability of their sense of bodily self [27]. Individuals who are susceptible to the rubber hand illusion are also more likely to have eating disorders [29], suggesting a possible link from self-objectification to disordered eating, through the mediating effect of body awareness, as measured by the rubber hand illusion. Given the importance of body awareness to self-objectification research, and the mixed results obtained with various questionnaire measures, we suggest that there is scope for the use of a well-validated physiological method, which has proved to be a reliable measure of body awareness in another area of psychology.

Biological psychology and more recent neuroscientific research have studied the self from the perspective of ‘interoception’, which has been defined as “the afferent information arising from within the body that affects the cognition or behaviour of an organism, with or without awareness” ([30] p 271). Interoception is a key component of recent influential models of the self. For example, our sense of ourselves as continuous and invariant over time is thought to be a function of the brain's continual representation of the interoceptive state of the body [31]. The self may, similarly, depend upon on the cortical re-integration of all consciously perceived ‘feelings’, in which interoception plays a major role [32].

The extent to which a person is aware of their internal bodily signals is known as ‘interoceptive awareness’. This varies between individuals and is generally assessed using a heartbeat perception task [33]–[34]. Recent studies have attempted to link this sensory perception of the body from *within*, measured by heartbeat perception, to the sensory perception of the body from the *outside*. For example, individuals with low interoceptive awareness have been shown to experience a stronger rubber hand illusion [35]. This suggests that the difficulty these people have in attending to their internal bodily signals is accompanied by a less accurate sense of their bodies as perceived externally. Conversely, when people with low interoceptive awareness pay attention to their bodies from an external perspective, during mirror self-observation, this enhances the accuracy with which they perceive their internal heartbeat cues [36].

These interactions between interoception and exteroception in self-processing resonate with reports that sufferers from anorexia nervosa, in whom preoccupation with the appearance of the external body is a key symptom [6], have lower interoceptive awareness than controls [37], which might reflect an inability to perceive the homeostatic interoceptive signals that set normal body weight. Individuals with psychosomatic complaints also have lower than average interoceptive awareness [38]. The blunted autonomic reactivity reported in such patients suggests that a deficiency in the detection of interoceptive signals could underlie their condition [39]. Taken together, the studies on the interaction between the body as perceived from within and the body as perceived from the outside, suggest that our degree of awareness of internal sensory states is related to our sensory perception of our bodies from a third-person perspective. These findings motivated the present study, which investigated the relationship between self-objectification and interoceptive awareness.

To the best of our knowledge, no one has previously used a heartbeat perception test as a measure of body awareness within self-objectification research. This is surprising, given Fredrickson and Roberts' original claim that self-objectification accounts for the poorer interoceptive awareness of women [16] as has often been reported in heartbeat perception studies [30], [40]. For example, experiments in the awareness of interoception have frequently (but not invariably) reported that women perform less well than men in accurate detection of both cardiac and gastric activity as well as respiratory resistance [30]. The inferior performance of women has previously been studied in terms of known confounds of interoceptive awareness, such as women's higher body fat, their generally lower physical fitness and differing cardiovascular variables, including smaller stroke volume of the heart [30]. In order to study the links between self-objectification and interoceptive awareness, independently of other known gender effects, we chose to investigate individual differences in women only.

Our experiment attempts to bridge the gap between the measures of self-awareness commonly used in the literature on objective self-focus and a psychophysiological measure of inner body awareness which is generally employed in research into interoception. We measured interoceptive awareness in young women, using a well-validated heartbeat tracking method [33], which has good test-retest reliability [38], [41] and correlates well with other measures of heartbeat detection [42]–[43]. Cardiac awareness correlates with awareness of other visceral signals, such as gastric or respiratory cues [18], [34], [44]. We compared our participants' interoceptive awareness with their scores on the Self-Objectification Questionnaire [19], the Self-Consciousness Scale [15] and the Body Consciousness Questionnaire [21].

Our principal hypothesis was that self-objectification would be predicted by interoceptive awareness and also by public self-consciousness and public body consciousness. We proposed interoceptive awareness as a predictor of self-objectification, in order to test the proposal that lower interoceptive awareness in women is related to self-objectification [16]. Public self-consciousness and public body consciousness were included in our model because both these scales refer to the self as perceived from a third-person perceptive and thus appear to have similarities to self-objectification [45].

The second purpose of our study was to test whether private body consciousness correlates with interoceptive awareness. Miller and colleagues found that both men and women high in private body consciousness reported more bodily changes, when they were secretly given caffeine, compared to those who were low in private body consciousness and also compared to participants who were high in private body consciousness but had received a placebo [21]. Their findings imply that private body consciousness is a good indicator of interoceptive awareness. Private body consciousness has frequently been used in the self-objectification literature as a measure of body awareness [22]–[24], [46] but with limited success. In the light of objectification research, we wished to test the relationship between private body consciousness and interoceptive awareness.

## Methods

### Ethics statement

The study was approved by the Department of Psychology Ethics Committee, Royal Holloway University of London. All participants were volunteers, who gave informed written consent and were free to withdraw at will.

### Participants

Participants were 50 female students at Royal Holloway University of London, aged 19–26 years, (mean = 21.04 years, SD = 1.33). Three participants were excluded for artefacts on their heartbeat traces and one for failing to comply with instructions on the Self-Objectification Questionnaire.

### Materials

#### The Self-Objectification Questionnaire (SOQ)

The Self-Objectification Questionnaire [19] measures the extent to which individuals view their bodies in observable, appearance-based (i.e. objectified) terms, versus non-observable competence-based terms. Participants are required to rank 10 body attributes by how important each is to their own physical self-concept, from 0 (for least impact) to 9 (greatest impact). Self-objectification scores are calculated by subtracting the summed ranks given to the 5 competence-based attributes (e.g. health, energy) from the summed ranks of the 5 appearance-based attributes (e.g. physical attractiveness, body measurements). Scores range from −25 to 25, with higher scores indicating greater emphasis on appearance, which is interpreted as greater self-objectification. The SOQ has good test-retest reliability (r = .92, cited in [45]).

#### Self-Consciousness Scale (SCS)

The Self-Consciousness Scale (SCS) [15] consists of three subscales, designed to measure self-focused attention. The Private Self-Consciousness subscale is made up of 10 items to assess the extent to which individuals focus on internal thought, sensations and feelings (e.g. “I'm always trying to figure myself out”). Public Self-Consciousness is measured by 7 questions referring to focusing on oneself as an object of an observer's scrutiny (e.g. “I usually worry about making a good impression”). There are 6 questions on the Social Anxiety subscale, which measures distress caused by interacting with other people (e.g. “I have trouble working when someone is watching me”). Participants respond on a 5-point Likert scale, ranging from 0 (extremely uncharacteristic of me) to 4 (extremely characteristic). Higher scores indicate greater self-consciousness/social anxiety. The three sub-scales appear to be relatively independent [15]. The SCS has fairly good reliability, with Cronbach's alpha ranging from .73 to .84 [4].

#### Body Consciousness Questionnaire (BCQ)

The Body-Consciousness Questionnaire [21] extends the concept of self-consciousness to awareness of the body. There are three subscales, which are Public Body Consciousness, Private Body Consciousness and Body Competence. The Private Body Consciousness subscale consists of 5 items designed to measure the tendency to focus on internal body sensations (e.g. “I am sensitive to internal body tensions”). Public Body Consciousness contains 6 questions to assess consciousness of the body as perceived by an observer (e.g. “I am very aware of my best and worst facial features”). Body Competence includes 5 items, which measure the individual's sense of body effectiveness. These Body Competence questions are somewhat similar to the competence-based questions in the Self-Objectification Questionnaire (e.g. “I'm better coordinated than most people”). Participants respond on a 5-point Likert scale, ranging from 0 (extremely uncharacteristic) to 4 (extremely characteristic). Higher scores represent greater body awareness/body competence. In a review of body awareness measures, the BPQ had high reliability and validity compared with other scales [26].

### Procedure

After giving informed consent, the participant's interoceptive awareness was measured. A piezo-electric pulse transducer was fitted to the left index finger and connected to a physiological data unit (26T PowerLab, AD Instruments), sampling at 1 kHz, which recorded the participant's pulse as derived electrical signal on a second PC, running LabChart6 software (AD Instruments). The Mental Tracking Method [33] was used, with a standard instruction [47] whereby participants were asked to concentrate hard and try to silently count their own heartbeats, simply by “listening” to their bodies, without taking their pulse. Instructions were presented over noise-attenuating headphones. The beginning and end of each heartbeat counting trial were cued by the words “go” and “stop”, presented audiovisually. After one training interval, there were three trial intervals, fully counterbalanced and always summing to 105s, which were selected from a set of intervals ranging from 20s to 55s. No feedback was given. Participants then completed the Self-Objectification Questionnaire, the Self-Consciousness Scale and the Body Consciousness Questionnaire.

## Results

LabChart6 was used to identify and count the number of R-wave peaks on the heartbeat trace, recorded for each participant in each trial, as well as to calculate the average heart rate for each trial [48]. Every heartbeat trace was visually inspected for artefacts and the number of R-wave peaks was recounted manually, if necessary. Three participants were excluded because artefacts created uncertainty about the number of their recorded beats.

Interoceptive awareness was calculated as {1/3 Σ [1 - (|recorded heartbeats – counted heartbeats|/recorded heartbeats)]} [33]. Higher scores indicate greater interoceptive awareness.

Descriptive statistics for all measures are shown in Table 1. Recorded values of the mean and standard deviation (SD) for all measures were similar to the values previously published by the authors of the various scales. Distributions for all measures were close to Gaussian, fulfilling an essential pre-condition for the use of multiple regression. In addition to using Private Self-Consciousness as a single measure, we split it into the subscales of Self-Reflectiveness and Internal State Awareness [49]. However, neither of these subscales was significantly correlated with either self-objectification or interoceptive awareness.

**Table 1:** Descriptive statistics for all measures.

	Min	Max	Mean	SD	Skew	Kurtosis
Interoceptive awareness	.22	.85	.59	.16	−.40	−.24
Self-objectification	−25	25	−1.65	13.80	.39	−.66
			c *1.09*	c *14.42*		
Private self-consciousness	14	36	24.83	4.79	−.12	−.10
			a *26.6*	a *5.1*		
Public self-consciousness	10	27	19.09	3.75	−.01	−.26
			a *19.3*	a *4.0*		
Social anxiety	4	23	13.04	4.57	.01	−.38
			a *12.8*	a *4.5*		
Private body consciousness	8	19	12.7	2.56	.02	−.29
			b *12.0*	b *3.3*		
Public body consciousness	9	22	16.41	3.0	−.02	−.25
			b *17.1*	b *3.3*		
Body competence	3	14	8.78	2.56	−.16	−.52
			b *10.0*	b *2.5*		

Note.

a Previously published mean and SD, n = 253 [15].

b Previously published mean and SD, n = 353 [21].

c Previously published mean and SD, n = 421 [19].

As shown in Table 2, there were significant inter-correlations amongst many of the measures we used. With two exceptions, these were close to the inter-correlations previously reported by the authors of the various scales. Unlike Miller and colleagues [21], however, we found no significant correlation between public self-consciousness and private body consciousness, nor between private self-consciousness and body competence.

**Table 2:** Correlations between the measures.

		SOQ	IA	Private SCS	Public SCS	Social anxiety	Private BCQ	Public BCQ	Body comp.
Interoceptive awareness (IA)	Correl.	−.31*							
	Sig.	.03							
Private self-consciousness	Correl.	.17	−.29						
(SCS)	Sig.	.26	.05						
Public self-consciousness	Correl.	.28	−.24	.36*					
	Sig.	.06	.11	.01					
	*Published correl.*			b.*23* **					
Social anxiety	Correl.	.26	−.26	.10	.30*				
	Sig.	.08	.09	.53	.04				
	*Published correl.*			b.*11*	b.*21* **				
Private body-consciousness	Correl.	−.18	−.04	.31*	−.08	−.24			
(BCQ)	Sig.	.23	.80	.04	.60	.10			
	*Published correl.*			a.*45* **	a.*28* **	a.*12*			
Public body-consciousness	Correl.	.47**	−.07	.28	.59**	.08	.09		
	Sig.	.000	.63	.06	.00	.59	.55		
	*Published correl.*			a.*33* **	a.*66* **	a.*12*	c.*37*		
Body competence	Correl.	−.14	.37*	.00	−.15	−.45**	−.35*	.05	
	Sig.	.36	.01	.98	.32	.00	.02	.72	
	*Published correl.*			a.*31* **	a.*09*	a *−.20*	c.*21*	c.*21*	
dSelf reflectiveness	Correl.	.17	−.28		.36*	.21	.25	.30*	−.13
	Sig.	.26	.06		.01	.16	.09	.05	.40
dInternal state awareness	Correl.	.02	−.11		.22	−.11	.31*	.15	.16
	Sig.	.88	.48		.14	.48	.04	.39	.27

Note.

a Previously published correlations for women, n = 353 [21].

b Previously published correlations for combined genders, n = 452 [15].

c Previously published correlations for combined genders, without probability values, n = 628 [21].

d Sub-division of the private self-consciousness scale [49].

* *p*<.05.

** *p*<.01.

All significance values are two-tailed.

**Figure 1:**
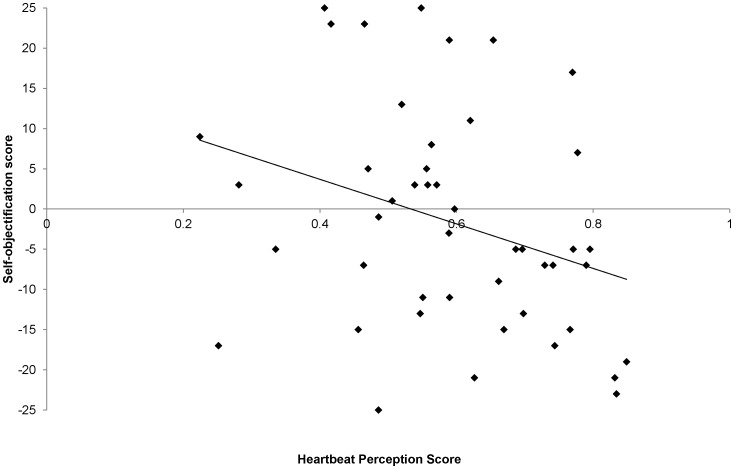
Scatter plot of self-objectification scores against interoceptive awareness.

We expected that self-objectification would be predicted by interoceptive awareness together with public self-consciousness and public body consciousness. Interoceptive awareness was significantly correlated with self-objectification, *r* = −.31, *p* = .03, (Figure 1). Multiple regression (entry method), with self-objectification as the dependent variable, showed that interoceptive awareness, public body consciousness, and private body consciousness together explained 31% of the variance in the self-objectification scores (Table 3). Neither public self-consciousness, nor any of the other questionnaire measures, made any significant contribution as a predictor. The three significant predictors were not inter-correlated, indicating an absence of multicollinearity within the model (see Table 2). No outlier analysis was performed.

**Table 3:** Multiple Regression (Entry method) with self-objectification as the Dependent Variable.

Predictor	Adjusted *R^2^*	Δ *R^2^*	*β*
*Step 1*			
Interoceptive awareness			−.31
(*sig.*)			*(.03* * *)*
	.08		
	*(.03* * *)*		
*Step 2*			
Interoceptive awareness			−.28
*(sig.)*			*(.03* * *)*
Public body-consciousness			.45
*(sig.)*			*(.001* ** *)*
	.27	.19	
	*(<.001* ** *)*		
*Step 3*			
Interoceptive awareness			−.29
*(sig.)*			*(.03* * *)*
Public body- consciousness			.47
*(sig.)*			(<.*001* **)
Private body-consciousness			−.23
*(sig.)*			*(.07* † *)*
	.31	.04	
	*(<.001* ** *)*		

Note.

† *p*<.1.

* *p*<.05.

** *p*<.01.

All significance values are two-tailed.

We recorded participants' average heart rates over the three heartbeat perception trials, as a proxy for physical arousal. Average heart rate was significantly correlated with social anxiety, *r* = .34, *p* = .02, and also with body competence, *r* = −.36, *p* = .02. However, there were no significant correlations between heart rate and the variables in our multiple regression (i.e. self-objectification, *r* = .12, *p* = .43, interoceptive awareness, *r* = −.16, *p* = .28, public body consciousness, *r* = −.05, *p* = .73, or private body consciousness, *r* = .14, *p* = .35), indicating that changes in participants' physical arousal are unlikely to have influenced our findings.

## Discussion

We measured interoceptive awareness in women students, using a well-validated heartbeat tracking method [33], which is common in interoception research. This was compared with scores on the Self-Objectification Scale [19], the Self-Consciousness Scale [15] and the Body Consciousness Scale [21]. Interoceptive awareness was significantly negatively correlated with self-objectification. Fredrickson and Roberts specifically claimed that women's poorer interoceptive awareness (measured by accuracy in their awareness of their heartbeats) is the result of self-objectification [16]. As far as we are aware, ours is the first study to confirm their prediction using a heartbeat perception method. We show that self-objectification in women was significantly predicted by a combination of interoceptive awareness, public body consciousness and private body consciousness, which together explained 31% of the variance in the self-objectification scores. Private body consciousness has frequently been used in self-objectification research as a measure of body awareness but in our study it was not significantly correlated with interoceptive awareness.

Not only were interoceptive awareness and private body consciousness uncorrelated in our study but they were also independent predictors of self-objectification (with low private body consciousness scores and low interoceptive awareness both predicting high self-objectification). This implies that the two measures tap into different, but perhaps complementary, aspects of internal body awareness. It may explain why many studies which have attempted to find paths from self-objectification to eating disorders and other negative outcomes, through the mediating effect of private body consciousness (as a measure of body awareness), have reported nonsignificant results [25]. Interoceptive awareness cannot measure important aspects of body awareness that are captured by self-report instruments, such as the individual's feelings about or responsiveness to bodily signals and her tendency to attend to or reflect on such sensations. However, we suggest that the use of a heartbeat perception measure of body awareness is likely to be more successful within objectification research, in view of this measure's applicability and validity in a wide range of research into interoception, emotion, alexithymia and anorexia.

Our findings also tested the assumption, frequently made in the self-objectification literature, that self-objectification is related to public self-consciousness and public body consciousness, because these two scales are designed to measure the individual's awareness of herself as perceived from a third-person perspective [45]. Public self-consciousness is a measure of an individual's thoughts and feelings about how other people perceive her (e.g. “I am concerned about what other people think of me”), whereas public body consciousness is specifically related to body awareness (e.g. “I'm concerned about my posture”). It was therefore more probable that the latter would be linked to self-objectification, which is defined as the tendency to perceive and judge one's body from a third-person perspective. We found that 27% of the variance in self-objectification scores was predicted by interoceptive awareness and public body consciousness, taken together. However, public self-consciousness was not significantly correlated with either interoceptive awareness or self-objectification in this study and did not contribute to our regression model.

Fredrickson and Roberts gave a purely attentional account for the link they expected would be found between self-objectification and interoceptive awareness, as measured by heartbeat perception [16]. They suggested that women who self-objectify are using up limited attentional resources on their bodies as perceived from a third-person perspective and so have less attention available for interoception. Pennebaker, in his ‘competition of cues’ hypothesis, argued similarly that when both internal and external sources of information are available, attention paid to one reduces attention paid to the other [50]. He made the crucial point that individuals direct their attention according to how they judge the salience of internal or external stimuli [51]. This implies that women who self-objectify tend to judge external stimuli (e.g. a real or imagined audience) as consistently more salient than their own interoceptive cues. It is not, however, clear why some women are more liable to self-objectify than others. Our results suggest that low interoceptive awareness may be a cause rather than an outcome of high self-objectification if women for whom internal stimuli are experienced, for innate or developmental reasons, as less salient, tend in consequence to direct their attention to their bodies from a third-person perspective. One potential explanation is that people differ in their ability to divide their attention between competing cues. Individuals with high interoceptive awareness perform significantly better on tests which require selective and divided attention [52]. According to our results, they will also tend to be low in self-objectification. More research is necessary to establish whether the ability to divide one's attention is a mediating variable, such that women with low interoceptive awareness not only tend to turn their attention outward but also have difficulty switching attention appropriately between salient internal and external cues.

The observed relation between low interoceptive awareness and high self-objectification, in our study, may be significant for emotional experience. High self-objectification is linked to negative affect [45] and to depressive symptoms [22]–[23], [46], [53]. Similarly, low interoceptive awareness is associated with moderate depression [54] and has been reported in a number of clinical conditions involving negative affect, such as anorexia [37] and somatoform disorders [38], as well as alexithymia - a disorder characterised by an inability to identify and describe one's emotions [55]. There is a wealth of evidence to suggest that individuals with high interoceptive awareness experience more emotional arousal, for the same objective bodily arousal, than people with low interoceptive awareness [56]–[60]. The results of our study, show that high self-objectification is predicted by low interoceptive awareness, implying that women who self-objectify are those who are relatively unaware of the interoceptive cues which are related to their emotions and who may also therefore experience emotion less intensely [25]. Such women may be vulnerable to clinical conditions associated with poor interoceptive awareness, such as anorexia, alexithymia and somatoform disorders. For example, poor emotional awareness, as measured by the Toronto Alexithymia Scale [61], has been shown to mediate between self-objectification and eating disorders, [46].

In our experiment, the second predictor of self-objectification was public body consciousness, which may also be a factor in the link between self-objectification and negative affect. In women, public body consciousness correlates with negative emotionality [21], which is with the tendency to get angry, upset or frightened (as measured by the emotionality subscale of the Emotionality, Activity, Sociability, Impulsivity, Temperament Scale [62]). As a predictor of self-objectification, public body consciousness may represent a measure of the tendency to experience negative affect.

The third significant predictor of self-objectification, in our regression equation, was private body consciousness, which predicted self-objectification independently of interoceptive awareness. These two measures did not correlate, which suggests that, while private body consciousness is significant for self-objectification, it is measuring something other than body awareness. Support for this idea is provided by the many studies that have attempted to use private body consciousness as a measure of internal body awareness in mediating between self-objectification and negative affect or eating disorders [25]. Success depends on the choice of instrument with which the body awareness is measured. Studies using private body consciousness generally report no effect [22], [24], [46]. One study [25] however, successfully used the Interoceptive Awareness scale of the Eating Disorders Inventory [7]. This scale is made up of items that assess awareness of emotions e.g. “When I am upset, I don't know if I am sad, frightened, or angry” and others that assess feelings of hunger and satiety e.g. “I get confused as to whether or not I'm hungry”. It was reported that the questions specific to hunger accounted for this instrument's success as a mediating variable [25]. In the light of the nonsignificant results obtained with most questionnaire measures of internal body awareness, it is surprising that physiological measures of have rarely been used in objectification research.

Our results imply that the ability to manipulate interoceptive awareness could benefit women who are high in self-objectification, as well as individuals with conditions where interoceptive awareness is abnormally low, such as anorexia, alexithymia, somatoform disorders and moderate depression. Interoceptive awareness has until recently been considered a robust trait variable because attempts to manipulate it have generally been ineffective [63]–[64]. However, an early study succeeded in altering interoceptive awareness by self-observation in a mirror [65], which is a typical means of heightening self-focus [66]. That experiment found that improvement in cardiac awareness was greater for participants low in public body consciousness (and who, according to our experiment, were probably also low in self-objectification). More recent research has shown that a mirror can be effective in raising interoceptive awareness in people who are poor heartbeat perceivers [36]. The combined results of these two studies, suggest that mirror self-observation can raise heartbeat perception in people with low interoceptive sensitivity. Whether higher interoceptive awareness would impact on self-objectification, and related conditions such as disordered eating, will depend on the complex interactions amongst mediating variables, such as Body Shame and Body Surveillance [23], [25], [53]. Taken together, the findings of the present study, in conjunction with experimental manipulations of interoception and the observation that patients with anorexia have lower interoceptive awareness, make interoception a central concept that should be considered in psychotherapeutic interventions of related disorders.

Objectification theory has assumed that poor interoceptive awareness is a function of self-objectification, as a consequence of consciously diverting attention to the ‘seen’ body, probably at the expense of attending to the inner body. However, we propose that low interoceptive awareness (together with public body consciousness) is a cause of self-objectification. Interoceptive awareness reflects the intensity with which people experience both positive and negative emotions [60] and is linked to the autonomic nervous system. Deficits in the autonomic system, which are associated with reduction in the inhibitory influence of prefrontal cortex, may account for the abnormally low interoceptive awareness reported in several clinical conditions [37], [39], [55]. Moreover, individuals who have high interoceptive awareness are less susceptible to the rubber hand illusion, which partially disengages autonomic regulation of hand temperature [67], and it has been reported that women who self-objectify are more prone to the rubber hand illusion [27]. An imbalance between sympathetic and parasympathetic activity in the autonomic system (assessed by skin conductance and cardiovascular measures, including heart rate variability) has been implicated in somatoform disorders [39], where sufferers are generally poor heartbeat perceivers. Conversely, good heartbeat perceivers show greater responsiveness of the autonomic system during emotional picture viewing and mental stress [68]. It has been proposed that autonomic imbalance, and associated hypoactivity of prefrontal cortex, underlie a number of other psychopathological conditions such as anxiety, depression and post traumatic stress [69]. It is significant in this context that, in our study, public body consciousness, which in women is associated with the tendency to experience specifically negative emotion, was an independent predictor of self-objectification. This suggests that participants who were highest in self-objectification may have had a deficit in parasympathetic activity, which could lead to negative feelings about their bodies and consequently to excessive self-monitoring [69]. Further research is required to establish how increased self-focus, for example through mirror self-observation, improves low interoceptive awareness. The effect may operate by making other aspects of the self more salient, thus enhancing attention to internal cues, or it may preferentially enhance parasympathetic activity.

Our experiment had certain limitations. Participants were well-educated young women, whose habitual tendencies to self-focus may not be typical of a broader population. However, our measures (and their inter-correlations) fell within the range of values previously reported for these instruments and appear representative. The primary purpose of the study was to investigate Fredrickson and Robert's claim that self-objectification is linked to heartbeat perception in women [16]. We therefore confined ourselves to the use of the Self-Objectification Questionnaire [19], Self-Consciousness Scales [15] and Body Consciousness Questionnaire [21], which have been widely used to study self-focus. Further research is required to establish the potential value of using heartbeat perception as a mediating variable between self-objectification and disordered eating. This would necessitate the use of measures such as Body Shame, and Body Surveillance [20], the Eating Disorders Inventory [7] and body mass index, which we chose not to include in this experiment.

This study is, we believe, the first to test Fredrickson and Robert's claim that interoceptive awareness in women, as measured by heartbeat perception, is negatively correlated with self-objectification [16]. We found that interoceptive awareness, together with public body consciousness and private body consciousness [21], accounted for 31% of the variance in scores in self-objectification. Despite its contribution as a predictor of self-objectification, private body consciousness was not significantly correlated with interoceptive awareness. This may account for the many experiments that have failed to find mediating effects between self-objectification and eating disorders or negative affect, when using private body consciousness as a measure of body awareness [25]. We propose that interoceptive awareness, as measured by heartbeat perception, has scope to be a more accurate and effective measure of body awareness within objectification research.
